# Serum CD109 levels reflect the node metastasis status in head and neck squamous cell carcinoma

**DOI:** 10.1002/cam4.3737

**Published:** 2021-02-09

**Authors:** Sumitaka Hagiwara, Eiichi Sasaki, Yasuhisa Hasegawa, Hidenori Suzuki, Daisuke Nishikawa, Shintaro Beppu, Hoshino Terada, Michi Sawabe, Masahide Takahashi, Nobuhiro Hanai

**Affiliations:** ^1^ Department of Head and Neck Surgery Aichi Cancer Center Hospital Nagoya Japan; ^2^ Department of Pathology and Molecular Diagnostics Aichi Cancer Center Hospital Nagoya Japan; ^3^ Department of Head and Neck Surgery ‐ Otolaryngology Asahi University Hospital Gifu Japan; ^4^ International Center for Cell and Gene Therapy Fujita Health University Toyoake Japan

**Keywords:** cancer biomarker, enzyme‐linked immunosorbent assay, head and neck squamous cell carcinoma, node metastasis, SCC antigen, serum CD109

## Abstract

**Background:**

Various biomarkers are being developed for the early diagnosis of cancer and for predicting its prognosis. The aim of this study is to evaluate the diagnostic significance of serum CD109 in head and neck squamous cell carcinoma (HNSCC).

**Methods:**

The serum CD109 levels in a total of 112 serum samples collected before and after surgery from 56 HNSCC patients were analyzed with an enzyme‐linked immunosorbent assay (ELISA). The clinical factor that showed a statistically significant association with both the preoperative serum CD109 level, and the CD109 index: which was defined as the ratio of the preoperative serum CD109 level to the postoperative serum CD109 level, were assessed. The correlations between the serum CD109 levels and lymph node density (LND), pathological features such as lymphatic invasion, and serum SCC antigen levels were also assessed.

**Results:**

The ELISA measurement revealed that preoperative serum CD109 levels were elevated in patients with node metastasis‐positive and stage IV disease, in comparison to those with node metastasis‐negative and Stage I+II+III disease, respectively. A multiple regression analysis indicated that serum CD109 level was significantly associated with the node metastasis status. A Spearman's rank correlation analysis also revealed a positive correlation between the preoperative serum CD109 level and LND. Furthermore, the probabilities of the overall and relapse‐free survival were significantly lower in patients with a preoperative serum CD109 level of ≥38.0 ng/ml and a CD109 index of ≥1.6, respectively, than in others. There was no significant correlation between the serum CD109 and SCC antigen levels.

**Conclusions:**

The serum CD109 levels were elevated in patients with advanced stage disease, reflecting the node metastasis status. CD109 in sera could be a novel prognostic marker for HNSCC involving lymph node metastasis.

## Graphical abstract

The serum CD109 levels in a total of 112 serum samples collected before and after surgery from 56 head and neck squamous cell carcinoma patients were analyzed with an enzyme‐linked immunosorbent assay. The serum CD109 levels were elevated in patients with advanced stage disease due to the node metastasis status, and the probability of overall and relapse‐free survival was significantly lower in patients with a high levels of serum CD109. CD109 in sera could be a novel prognostic marker for head and neck squamous cell carcinoma involving lymph node metastasis.

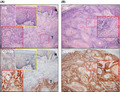

## 1 INTRODUCTION

Many patients with head and neck cancer are diagnosed with advanced‐stage lesions despite having several clinical symptoms and the ease of clinical examination by inspection and by palpation. One of the reasons for this is that in addition to the tumor size, the node metastasis status is a critical factor for staging. Although improvements in diagnostic imaging modalities, such as contrast‐enhanced computed tomography and positron emission tomography have contributed to evaluating the patient's clinical status, patients who are clinically diagnosed as node‐negative are sometimes found to be node positive based on pathological examinations after surgery. Recently, numerous blood cancer biomarkers have been developed and applied, including CEA in lung and gastrointestinal cancer, PSA in prostate cancer, and CA19‐9 in pancreatic cancer. Squamous cell carcinoma (SCC) accounts for the majority of malignant neoplasms in the head and neck region, and with the exception of squamous cell carcinoma antigen (SCC‐Ag), there are few useful biomarkers for this disease. SCC‐Ag is originally purified in human cervical SCC, and can be detected in patients with cervical SCC, but not in healthy women.^1^ However, while the upregulation of serum SCC‐Ag level would indicate the presence of SCC, it does not always reflect the malignant status. In head and neck squamous cell carcinoma (HNSCC), it was reported that the rate of SCC‐Ag positivity ranged widely between 20% and 78%.^2^

**FIGURE 1:**
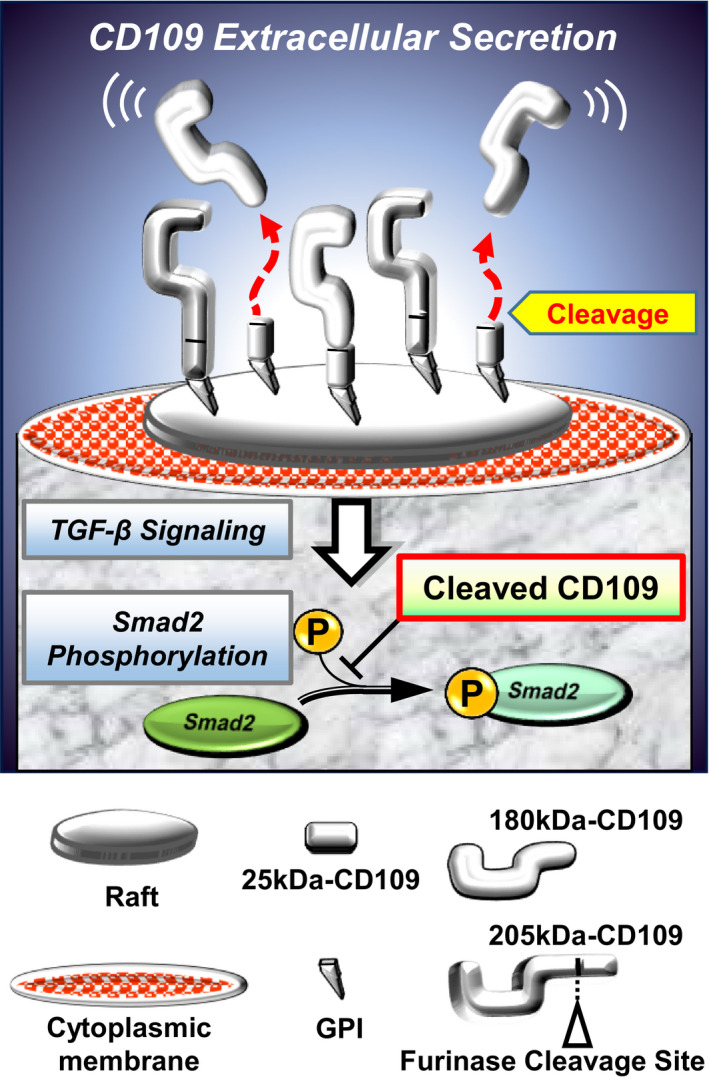
An illustration showing the secretion of CD109. CD109, GPI‐anchored cell surface glycoprotein, is composed of 180 and 25 kDa fragments. 205 kDa‐CD109 is degraded at the furinase cleavage site, and 180 kDa‐CD109 is secreted into the extracellular fluid. In addition, degraded CD109 negatively regulates TGF‐β/Smad signaling

CD109, TGF‐β/smad signaling modulator, is a glycosylphosphatidylinositol (GPI)‐anchored cell surface glycoprotein and a member of the α2‐macroglobulin (α2M)‐C3, C4 and C5 family. This molecule was first identified as a 170–180 kDa cell‐surface antigen and was isolated from the KG1a lymphoid/myeloid cell line.^3^, ^4^, ^5^ CD109 protein has an N‐terminal signal sequence (amino acids [aa.]1–21), a furinase cleavage site (FCS, aa.1270–1273) and a GPI‐anchor addition site (aa.1420–1445). Our study group previously reported that the *CD109* transcript expression levels were remarkably upregulated in SCCs of the esophagus, lung and cervix.^6^, ^7^ Immunohistochemical analyses using clinical specimens of lung and oral cavity revealed that high expression of CD109 was more frequently detected in SCCs than in normal tissue.^8^, ^9^ In addition, our *in vitro* studies using CD109 overexpressing cell‐lines indicated that N‐terminal 180 kDa‐CD109 fragments cleaved at FCS of full‐length 205 kDa‐CD109 underwent extracellular secretion as a soluble form (Figure 1).^10^ Furthermore, we found that 180 kDa soluble CD109, which is also secreted as a component of exosomes, could be detected in the sera of tumor xenografted mice.^11^, ^12^

Our previous data suggested that soluble CD109 would be present in the sera of SCC patients, and that it may have a potential application as a novel biomarker, similar to SCC‐Ag. In this study, we performed a quantitative evaluation of CD109 by an enzyme‐linked immunosorbent assay (ELISA) using serum samples collected from HNSCC patients and demonstrated the clinical significance of its expression.

## 2 MATERIALS AND METHODS

### 2.1 Patients and serum samples

The study population included patients with SCC in the head and neck region who underwent surgical treatment at the department of head and neck surgery of Aichi Cancer Center Hospital from September 2014 till December 2016. This study was performed retrospectively, and cases for which sera had been collected at both preoperative and postoperative periods were selected. Cases with distant metastasis, or in which neoadjuvant chemotherapy or preoperative radiotherapy (at an appropriate site) had been performed were excluded. Preoperative samples were collected within one month before surgery as part of a screening test, and postoperative samples were collected as part of a clinical follow‐up test at the cancer‐free phase. Each sample was stored at −80°C in the clinical laboratory of our hospital, without being thawed until measurements were performed.

### 2.2 Stratification of clinical features

Fifty‐six cases of HNSCC were stratified according to clinical information about sex, age, tumor locus, pathological T status (pT status), pathological N status (pN status), pathological Stage (pStage), histopathology, and history of drinking and smoking. pT, pN and pStage were determined according to the 8th edition of the UICC’s TNM classification system (UICC 8th edition). The clinical features of the patients are summarized in Table 1, and the numbers of each clinical factor are displayed according to the tumor locus. In oropharyngeal cancer, a retrospective pathological examination using p16 immunohistochemistry (CINtec Histology Kit, clone E6H4; Ventana) and GP5+/GP6+ consensus primers for the L1 region (150‐bp product) revealed that two of seven cases were human papillomavirus (HPV)‐positive SCC.

**TABLE 1:** The clinical features and number of patients with each clinical factor

				Tumor locus^$^					
Clinical factors		Total		Oral cavity			Pharynx and others		
				Tongue	Gingiva	Others (1)	Oropharynx^¶^	Hypopharynx	Others (2)
Total	n	56		20	11	6	7	7	5
	(%)			(35.7)	(19.6)	(10.7)	(12.5)	(12.5)	(8.9)
Sex									
Male		35		9	6	5	4	6	5
Female		21		11	5	1	3	1	0
Mean age									
Years		65.6		62.1	72.6	63.7	57.1	71.1	71.7
Range		30–87		30–84	59–82	46–70	36–78	58–87	64–80
pT status									
T1		17		8	3	1	1	1	3
T2		20		5	2	3	5	4	1
T3		9		6	0	0	1	1	1
T4		10		1	6	2	0	1	0
pN status									
N (−)	ND(−)^#^	20		6	4	3	3	2	2
	N0	20		11	5	1	0	1	2
N (+)	N1	5		1	0	1	2	1	0
	N2	7		2	2	1	1	1	0
	N3	4		0	0	0	1	2	1
pStage									
Exclude 20 ND(−) Cases									
I		5		2	1	0	1	0	1
II		7		4	1	0	0	1	1
III		8		5	0	1	1	1	0
IV		16		3	5	2	2	3	1
Histopathology (SCC differentiation)									
Well			29	10	9	2	3	1	4
Moderate			19	7	1	4	3	3	1
Poor			8	3	1	0	1	3	0
Drinking									
Never			22	10	4	3	1	3	1
Current / Ever			34	10	7	3	6	4	4
Smoking									
Never			22	10	6	3	2	1	0
Current / Ever			34	10	5	3	5	6	5

# ND(−) indicates the cases that did not undergo neck dissection.

$ Others (1): four buccal mucosa cases, and two cases of the floor of the mouth. Others (2): four larynx cases, and one maxillary sinus case.

¶ Two of seven cases were human papillomavirus (HPV)‐positive SCC cases.

### 2.3 Enzyme‐linked immunosorbent assay

An enzyme‐linked immunosorbent assay (ELISA) test kit for CD109 (Cat No.: SEB458Hu) was purchased from Cloud‐Clone Corp. This kit is a sandwich enzyme immunoassay for quantitative measurement in human tissue homogenates, cell lysates and other biological fluids. For a reliable data analysis, an external biochemical laboratory was commissioned to perform the measurement of all serum samples, and analyses were performed according to the instruction manual. All samples were adjusted to 50‐fold dilution and examined using two wells. The color change with an enzyme‐substrate reaction was measured spectrophotometrically at a wavelength of 450 nm. The average of duplicate measurements was used as the serum CD109 value.

### 2.4 Immunohistochemistry

Anti‐CD109 mouse monoclonal antibody (Cat No.: sc‐271085) was purchased from Santa Cruz Biotechnology. Immunohistochemical staining was performed on formalin‐fixed paraffin‐embedded sections following standard procedures on Dako automated instruments, using 50‐fold diluted anti‐CD109 antibody. Nuclear counterstaining was performed using hematoxylin. Appropriate staining was defined based on the presence of immunoreactivity in both the cytoplasm and plasma membrane. The CD109 expression in tumor tissues was judged as positive when ≥10% of the tumor region was stained.

### 2.5 Lymph node density (LND)

The LND was calculated as the ratio of the number of metastasis‐positive lymph nodes to the total number of excised lymph nodes. The LND was pathologically investigated for all 36 patients who underwent neck dissection.

### 2.6 Clinical ethics and privacy protection

This study was conducted with the approval of the clinical ethics committee of Aichi Cancer Center Hospital. The personal information, including the clinical profile of each sample, was anonymized for connection using correspondence tables. In brief, personally identifying information was removed when the samples were transported and was connected again after receiving the data sheet from laboratory.

### 2.7 Statistical analyses

Two‐group comparisons of preoperative or postoperative samples were carried out for each clinical factor using Student's *t*‐test or Welch's *t*‐test. The comparisons between the clinical factors of the preoperative and postoperative groups were performed using paired *t*‐tests. To identify the clinical factors that were significantly associated with the serum CD109 levels, a multiple regression analysis was performed after the extraction of clinical factors that showed *p*‐values of < 0.5 in the two‐group comparisons. The Kaplan‐Meier method with a log‐rank test was used for the survival analysis for both overall survival (OS) and relapse‐free survival (RFS). RFS was defined as the time from the operation until first recurrence including distant metastasis or death due to any cause. A Spearman's rank correlation analysis was performed to investigate the correlation between the serum CD109 level, LND, and serum SCC‐Ag level. *p* values of <0.05 were considered as significant. All statistical analyses were performed using the EZR software program (version 1.41).^13^

## 3 RESULTS

### 3.1 The distribution of the serum CD109 levels

**FIGURE 2:**
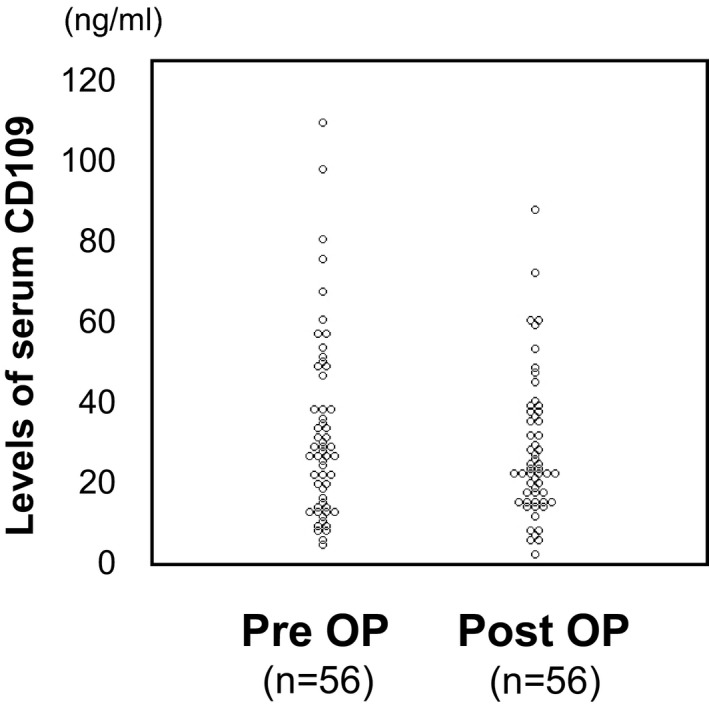
The distribution of serum CD109 levels in the perioperative period. “Pre OP” indicates the preoperative serum CD109 levels; “Post OP” indicates the postoperative serum CD109 levels

Dot plots of the serum CD109 levels in a total of 112 samples are shown in Figure 2. The mean preoperative and postoperative serum CD109 levels in all 56 cases were 32.24 ± 22.39 [mean ±standard deviation (SD)] ng/ml and 27.89 ± 17.24 ng/ml, respectively. A paired *t*‐test revealed that the mean postoperative serum CD109 level was significantly reduced in comparison to the mean preoperative serum CD109 level (*p* < 0.05) (Table 2). Tumor resection seemed to be an important event for reducing the serum CD109 level.

**TABLE 2:** Two‐group comparisons of the perioperative serum CD109 levels for each clinical factor

			Mean value (ng/ml)				*P*‐value Pre OP vs Post OP (Paired *t*‐test)
Clinical factors		n	Pre OP	*P*‐value (*t*‐test)	Post OP	*P*‐value (*t*‐test)	
Total		56	**32.24**	―	**27.89**	―	<*0*.*05* *
Sex	Male	35	**33.39**	*N*.*S*.	**28.87**	*N*.*S*.	*N*.*S*.
	Female	21	**30.34**		**26.27**		*N*.*S*.
Age	≥ 66	31	**32.96**	*N*.*S*.	**29.10**	*N*.*S*.	*N*.*S*.
	< 66	25	**31.35**		**26.40**		*N*.*S*.
Tumor locus	Oral cavity	37	**30.35**	*N*.*S*.	**26.31**	*N*.*S*.	*N*.*S*.
	Pharynx / Others	19	**35.92**		**30.98**		*N*.*S*.
pT status	T1+T2	37	**28.67**	*N*.*S*.	**26.76**	*N*.*S*.	*N*.*S*.
	T3+T4	19	**39.21**		**30.10**		*0*.*034* *
pN status	N(‐)	40	**27.66**	*0*.*047* *	**27.79**	*N*.*S*.	*N*.*S*.
	N(+)	16	**43.70**		**28.15**		<*0*.*005* *
pStage^#^	I+II+III	20	**27.52**	*0*.*026* *	**26.45**	*N*.*S*.	*N*.*S*.
	IV	16	**46.02**		**29.42**		<*0*.*004* *
Histopathology	Well	29	**35.41**	*N*.*S*.	**30.64**	*N*.*S*.	*N*.*S*.
	Moderate / Poor	27	**28.85**		**24.94**		*N*.*S*.
Drinking	Never	22	**31.81**	*N*.*S*.	**24.90**	*N*.*S*.	*N*.*S*.
	Current / Ever	34	**32.53**		**29.83**		*N*.*S*.
Smoking	Never	22	**34.41**	*N*.*S*.	**27.15**	*N*.*S*.	*N*.*S*.
	Current / Ever	34	**30.84**		**28.37**		*N*.*S*.

Abbreviations: N.S.: not significant; Pre OP, preoperative serum; Post OP, postoperative serum.

# Exclude 20 ND(−) cases.

* Statistical significance.

### 3.2 Two‐group comparisons of the preoperative and postoperative serum CD109 levels in each clinical factor

**FIGURE 3:**
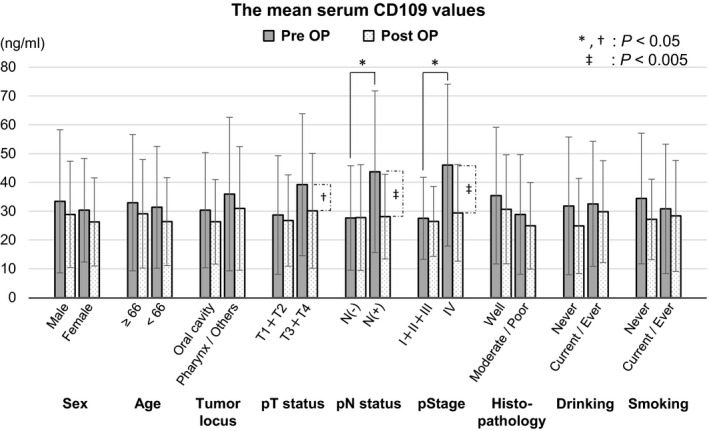
Two‐group comparisons of the perioperative serum CD109 levels in each clinical factor. “Pre OP” (gray bar charts) and “Post OP” (doted white bar charts) indicate preoperative serum CD109 levels and postoperative serum CD109 levels, respectively. Each error bar indicates the standard deviation. “N(−)” and “N(+)” indicate node metastasis‐negative, including patients who did not undergo neck dissection, and the node metastasis‐positive, respectively. *: Statistical significance in preoperative two‐group comparisons using Student's or Welch's *t*‐test. †, ‡: Statistical significance in preoperative to postoperative alteration using paired *t*‐test

According to 2‐group comparisons, the mean preoperative serum CD109 values in the node metastasis‐positive (pN status) group (43.70 ng/ml) and the UICC 8th edition Stage IV (pStage) group (46.02 ng/ml) were significantly higher than in the node metastasis‐negative (27.66 ng/ml) and the UICC 8th edition Stage I+II+III (27.52 ng/ml) groups, respectively (Table 2 and Figure 3). Two‐group comparisons of other preoperative clinical factors showed no statistically significant differences.

Two‐group comparisons of the mean postoperative serum CD109 values indicated no significant differences for any clinical factors. As shown in Table 2 and Figure 3, the mean postoperative CD109 values in the UICC 8th edition T3+T4 group, the node metastasis‐positive group, and the UICC 8th edition Stage IV group were significantly reduced, which were almost the same levels as those in the T1+T2, the node metastasis‐negative, and the Stage I+II+III groups, respectively. The preoperative serum CD109 level was increased in the patients with advanced‐stage disease.

### 3.3 Two‐group comparisons of the CD109 index in each clinical factor

Next, the CD109 index, which was defined as the ratio of the preoperative serum CD109 level to the postoperative serum CD109 level was determined in each case, and its clinical significance was investigated. This index would make it possible to evaluate the relative changes in the perioperative serum CD109 levels and to correct the variability in each patient. The mean CD109 index in 56 cases was 1.25 (SD, 0.59). Two‐group comparisons according to the CD109 index were performed using Student's *t*‐test for each clinical factor. As shown in Table 3, statistically significant differences were detected in pN status and in pStage (*p* < 0.004 and *p* < 0.003, respectively), but were not detected in other clinical factors, including the pT status. Regarding both the pN status and pStage, the 2‐group comparisons’ *p*‐values of the CD109 index were remarkably lower than those of the preoperative serum CD109 levels.

**TABLE 3:** Two‐group comparisons of the CD109 index for each clinical factor

Clinical factors		n	CD109 index		*P*‐value (*t*‐test)
			Mean	SD	
Total		56	**1.25**	0.59	―
Sex	Male	35	**1.21**	0.57	*N*.*S*.
	Female	21	**1.32**	0.63	
Age	≥66	31	**1.24**	0.61	*N*.*S*.
	<66	25	**1.27**	0.59	
Tumor locus					
	Oral cavity	37	**1.24**	0.58	*N*.*S*.
	Pharynx/Others	19	**1.28**	0.62	
pT status	T1+T2	37	**1.17**	0.56	*N*.*S*.
	T3+T4	19	**1.41**	0.65	
pN status	N(−)	40	**1.11**	0.54	<*0*.*004* *
	N(+)	16	**1.60**	0.58	
pStage^#^	I+II+III	20	**1.09**	0.43	<*0*.*003* *
	IV	16	**1.66**	0.64	
Histopathology					
	Well	29	**1.22**	0.59	*N*.*S*.
	Moderate/Poor	27	**1.29**	0.60	
Drinking	Never	22	**1.38**	0.66	*N*.*S*.
	Current/Ever	34	**1.17**	0.54	
Smoking	Never	22	**1.32**	0.65	*N*.*S*.
	Current / Ever	34	**1.21**	0.55	

Abbreviations: N.S.: not significant; SD: standard deviation.

# Exclude 20 ND(−) cases.

* Statistical significance.

### 3.4 The multiple regression analysis of factors associated with the preoperative serum CD109 level and the CD109 index

To identify clinical factors that were independently associated with both the preoperative serum CD109 level and the CD109 index, a multiple regression analysis was performed. pStage was excluded as a confounding factor. Tumor locus, pT status, pN status, and histopathology were selected as explanatory variables for the analysis of factors associated with the preoperative serum CD109 level, whereas the pT status, pN status, drinking history, and smoking history were selected for the analysis of factors associated with the CD109 index. In the multiple regression analyses, the pN status was the only clinical factor that showed a statistically significant association with both the preoperative serum CD109 level and the CD109 index (Table 4). Regarding the pN status of node metastasis‐negative and ‐positive cases, the cut‐off values were determined for both the preoperative serum CD109 level and the CD109 index using a Receiver Operating Characteristic (ROC) analysis. When the cut‐off value of the preoperative serum CD109 level was set to 26.3 ng/ml (sensitivity: 0.875, specificity: 0.575, and Area Under the Curve [AUC]: 0.695), Fisher's exact test significantly distinguished the node‐positive cases in the following three groups: ND (‐) which was the group of not performed neck dissection, pN0, and pN 1–3 (*p* = 0.007). When the cut‐off value of the CD109 index was set to 1.15 (sensitivity: 0.812, specificity: 0.625, and AUC: 0.750), Fisher's exact test also significantly distinguished the node‐positive cases (*p* < 0.01) (Table 5). These data suggest that the serum CD109 level reflects the node metastasis status in HNSCC patients.

**TABLE 4:** Result of a multiple regression analysis of factors associated with the preoperative serum CD109 level and CD109 index

*p* < 0.5 Clinical features in *t*‐test	Preoperative serum CD109 level		CD109 index	
	*P*‐value		*P*‐value	
	*t*‐test	Multiple regression	*t*‐test	Multiple regression
Tumor locus	0.383	**0.557**	>0.5	**—**
pT status	0.096	**0.303**	0.167	**0.479**
pN status	0.047*	**0.037** *	<0.004*	**0.013** *
Histopathology	0.277	**0.156**	>0.5	**—**
Drinking	>0.5	**—**	0.205	**0.595**
Smoking	>0.5	**—**	0.487	**0.868**

* Statistical significance.

**TABLE 5:** Fisher's exact test of the correlation between the serum CD109 level and node metastasis

	Group	pN status: n (Total 56 cases)			*P*‐value
		ND(−)^#^: 20	pN0: 20	pN1‐3: 16	
Pre OP (ng/ml)	≥26.3	8	9	14	0.007*
	<26.3	12	11	2	
CD109 index	≥1.15	6	10	13	<0.01*
	<1.15	14	10	3	

Pre OP: preoperative serum CD109 level.

# ND(−) indicates the cases that did not undergo neck dissection.

* Statistical significance.

### 3.5 The CD109 expression in node metastasis‐positive HNSCC tissues

**FIGURE 4:**
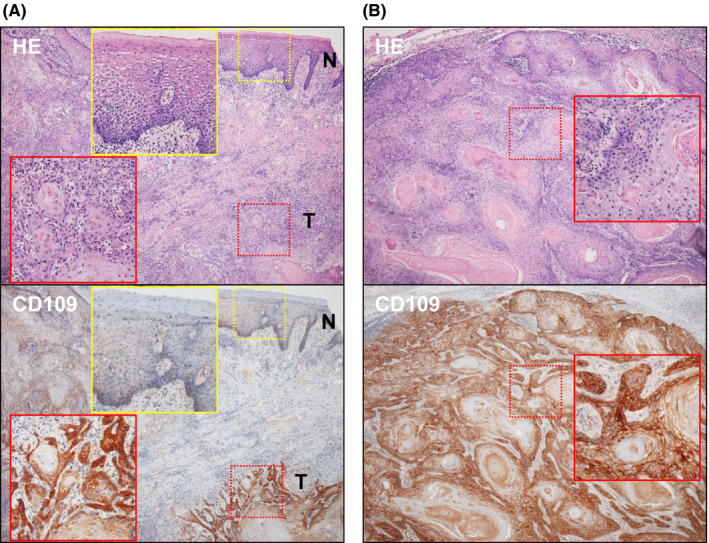
Immunohistochemistry in a representative case of node metastasis‐positive HNSCC (well‐differentiated tongue SCC). (A) HE staining and immunohistochemical staining of CD109 in the primary tumor site. ‘N’ and ‘T’ indicate the normal squamous epithelium and the invasive tumor lesion, respectively. (B) HE staining and immunohistochemical staining of CD109 in the metastatic lymph node. The insets in each panel are the high power of ×200 magnification. The normal squamous epithelium was almost completely negative for CD109. CD109‐positive cells were observed in not only the primary tumor but also the metastatic node

To confirm the CD109 expression in surgically resected tissues, immunohistochemistry with anti‐CD109 antibody was performed in seven node metastasis‐positive cases with paraffin‐embedded specimens that were suitable for immunostaining. As shown in Figure 4, both the primary tumor lesion and the metastatic lymph node were positive for CD109 protein in all seven cases, while the normal squamous epithelium was almost negative. CD109 was highly expressed in invasive tumor nests and in malignant cells surrounding the keratinized region.

**FIGURE 5:**
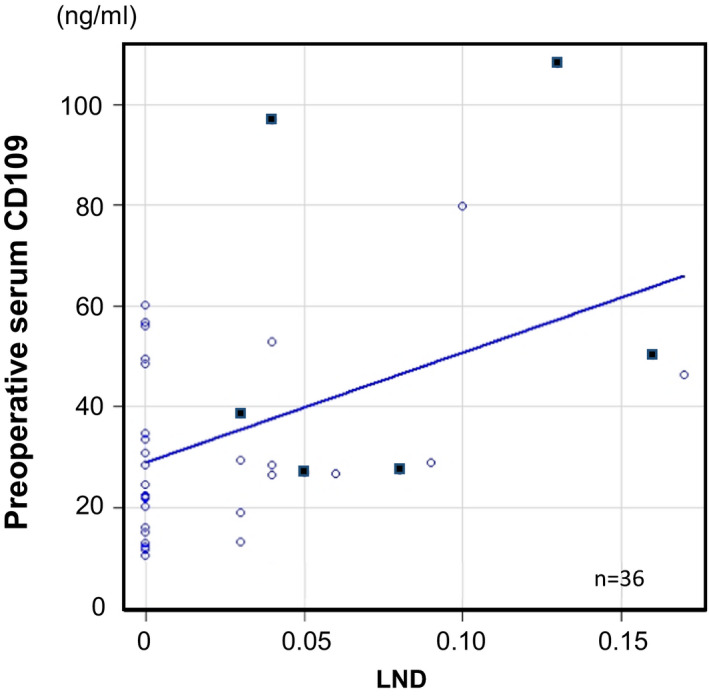
Result of a Spearman's rank correlation analysis of the association between the serum CD109 level and LND. A significant positive relationship was observed among the 36 analyzed cases. The black dots indicate the cases with extranodal extension

In addition, the correlation between the preoperative serum CD109 and LND values in each case was examined using Spearman's rank correlation coefficient. As shown in Figure 5, the analysis of 36 samples revealed a slight positive correlation between the preoperative serum CD109 level and LND with statistical significance (*rs *= 0.368 *p* = 0.027). The CD109 expression in these pathological analyses suggests that serum CD109 is associated with advanced HNSCC involving lymph node metastasis.

The correlations between the preoperative serum CD109 level and pathological features, such as lymphatic invasion (ly: −/+), vascular invasion (v: −/+), perineural invasion (pn: −/+), were also assessed in a total of 56 cases. Two‐group comparisons of the mean preoperative serum CD109 values using Student's *t*‐test indicated no significant differences in any pathological features (data not shown).

### 3.6 Serum CD109 predicts a poor prognosis in HNSCC

**FIGURE 6:**
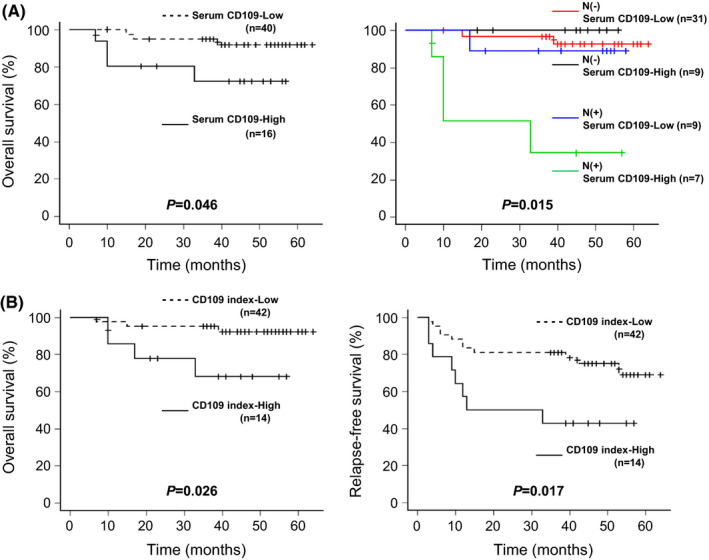
The Kaplan‐Meier analysis with the log‐rank test for both overall survival (OS) and relapse‐free survival (RFS). The OS rate was significantly lower in the group with a preoperative serum CD109 level of ≥38.0 µg/ml: “Serum CD109‐High” (A), and in the group with a CD109 index of ≥1.6: “CD109 index‐High” (B). “N(−)” and “N(+)” indicate node metastasis‐negative, including the patients who did not undergo neck dissection, and the node metastasis‐positive, respectively. The subgroup with both “Serum CD109‐High” and “N(+)” factors showed an extremely poor prognosis. The RFS rate was also significantly lower in the group with “CD109 index‐High”

Next, a Kaplan‐Meier method survival analysis was performed to evaluate the impact of the serum CD109 level on the prognosis. The mean observation period in 56 cases was 41.7 months (range, 7 to 64 months). The cut‐off values of the preoperative serum CD109 level and the CD109 index were calculated by an ROC analysis based on the OS as 38.0 ng/ml and 1.6, respectively. As shown in Figure 6A, the OS rate was significantly lower in patients with a preoperative serum CD109 level of ≥38.0 ng/ml (*p* = 0.046). The subgroup with both a preoperative serum CD109 level of ≥38.0 ng/ml and a node metastasis positivity showed the lowest OS rate (*p* = 0.015). As shown in Figure 6B, not only the OS rate but also the RFS rate was significantly lower in patients with a CD109 index of ≥1.6 than in others (*p* = 0.026 and *p* = 0.017, respectively). These results suggest that the preoperative serum CD109 levels can detect poor prognostic HNSCC cases with node metastasis and that the CD109 index can predict patients who are at risk of locoregional recurrence or distant metastasis.

### 3.7 Relationship between the serum levels of CD109 and SCC‐Ag

**FIGURE 7:**
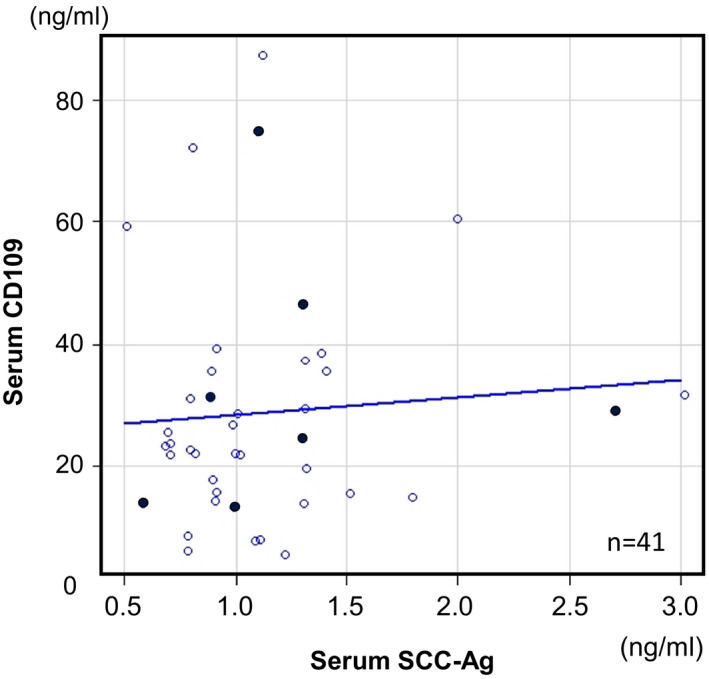
Spearman's rank correlation analysis of the association between the serum levels of CD109 and SCC‐Ag. No significant positive relationship was observed in any of the 41 analyzed cases. The black dots and white dots indicate the preoperative cases and the postoperative cases, respectively

The relationship between the serum level of CD109 and that of SCC‐Ag was examined. The samples whose serum SCC‐Ag value was measured as part of the clinical examinations at our hospital: seven preoperative cases and 34 postoperative cases in a total of 112 samples: were included. The serum CD109 and SCC‐Ag levels in each sample were analyzed using Spearman's rank correlation coefficient. As shown in Figure 7, the analysis of 41 samples revealed no significant correlation between the serum CD109 and SCC‐Ag levels (*rs *= 0.132, *p* = 0.409). There were several cases in which the preoperative serum CD109 level was high, even when the preoperative SCC‐Ag level was normal (<1.5 ng/ml). These cases might suggest that serum CD109 level could help identify a malignant status in cases that were not identified by SCC‐Ag.

## 4 DISCUSSION

As a primary finding of this study, the serum CD109 levels of HNSCC patients (as determined by an ELISA) were significantly elevated in advanced cases (defined according to the pN/pStage). Since 2004 when Hashimoto *et al*. reported the expression of CD109 in human cancer,^6^ our group has earnestly investigated the immunohistochemical CD109 expression of tumor tissues, including that in lung and oral cancers,^8^, ^9^, ^14^, ^15^ and has performed biological analyses using *CD109* transfected cells or transgenic mice.^10^, ^11^, ^12^, ^16^ Although few reports have described the use of serum samples, recently, many research groups have focused on CD109 and have reported the upregulation of its immunohistochemical expression in multiple types of malignant tumors.^17^, ^18^, ^19^, ^20^, ^21^, ^22^, ^23^, ^24^ Given the results of our previous studies, one potential secretory origin may be epithelial cells with uncontrolled proliferation. As the precedent for analyzing serum CD109 levels by ELISA, a previous study demonstrated that CD109 levels in patients with non‐small‐cell lung cancer were elevated in comparison to normal subjects.^25^ In our ELISA‐based analysis of HNSCC, a CD109 level above the cut‐off value (preoperative CD109 level of 38.0 ng/ml and CD109 index of 1.6) was associated with a poor prognosis and lymph node metastasis.

Similarly to a previous report that demonstrated that CD109 could inhibit TGF‐β signaling by the direct modulation of receptor activity,^26^ we also clarified that CD109 promotes cell proliferation due to the negative regulation of TGF‐β1/Smad2 signaling.^9^, ^10^ On the other hand, it was reported that CD109 released from human bone marrow mesenchymal stem cells downregulates the TGF‐β‐induced epithelial‐mesenchymal transition (EMT) and stemness of SCC,^27^ or that the expression of CD109 is inversely correlated with TGF‐β signaling and the EMT in cultured SCC cells.^28^ Controversially, Zhang *et al*. demonstrated—using a glioblastoma cell line—that CD109 attenuates TGF‐β1 signaling, whereas it enhances EGF signaling, cell migration and invasion.^29^ Sunagawa *et al*. reported that CD109 deficiency suppresses skin tumorigenesis by enhancing TGF‐β/Smad/Nrf2 pathway activity in mice.^30^ At the chromosome level, the amplification of a specific region associated with the overexpression of *CD109 *has been confirmed in head and neck cancer cell lines.^31^, ^32^ As described above, several biological studies support the results of this study, which demonstrated that the serum levels of CD109 were high in patients with advanced HNSCC.

SCC‐Ag, which is an excellent biomarker for SCC, is currently applied as a diagnostic marker in the clinical setting. The serum level of SCC‐Ag is reported to be elevated in HNSCC patients and its level is reported to be reduced after cancer treatment.^33^, ^34^ This biomarker was previously reported to show a strong association with the survival rate, and serial observation of serum SCC‐Ag after treatment would alert physicians to the recurrence of HNSCC.^35^, ^36^ Several studies have demonstrated that the combined evaluation of SCC‐Ag and other biological indicators would contribute to improve sensitivity in the detection of lymph node metastasis and tumor recurrence or act as a predictor of these states.^37^, ^38^, ^39^, ^40^, ^41^ Adel *et al*. reported that concurrent high preoperative serum levels of SCC‐Ag and C‐reactive protein exhibited a positive correlation with LND and were associated with a poor prognosis in oral SCC.^40^ On the contrary, it is also reported that SCC‐Ag is not effective in the diagnosis of early‐stage HNSCC,^42^ and that it does not predict OS in oropharyngeal cancer.^43^ A meta‐analysis of a total of 1901 cases in 11 eligible studies reviewed by Travassos *et al*. demonstrated that elevated SCC‐Ag levels are significantly correlated with males and TNM stage, and may not be used as a predictive marker for OS or DFS in HNSCC patients.^2^ Our study shows a correlation between the serum CD109 level and LND. The LND was previously reported to be a prognostic indicator of head and neck malignancy including major salivary gland carcinoma.^44^, ^45^, ^46^, ^47^ If the evaluation of SCC‐Ag levels alone is unsuitable for the early diagnosis of HNSCC or predicting a poor prognosis in HNSCC, the prognostic characteristics of CD109 might be responsible for the lack of correlation between the serum levels of CD109 and SCC‐Ag.

Our study was conducted as a retrospective study, and serum samples were not collected from healthy controls in the same survey period. Thus, the clinical significance of the serum CD109 level under a cancer‐bearing status was assessed according to the ratio of the preoperative value to the postoperative value in each case. In addition, cases that received preoperative treatment or for which appropriate serum samples were not available were excluded from this analysis; thus, the study population was limited to 56 cases. Given that sera may contain secreted CD109 derived from platelets or vascular endothelial cells,^48^, ^49^ the study design should have included a control group of healthy subjects. Although further clinical studies of CD109 are required to improve these limitations, CD109 is expected to be a novel prognostic marker or therapeutic target for HNSCC in cases with lymph node metastasis.

## CONFLICT OF INTEREST

The authors declare no conflicts of interest.

## AUTHOR CONTRIBUTIONS

Conceptualization, data acquisition, investigation and formal analysis, writing and editing: Sumitaka Hagiwara. Collection of clinical samples, pathological investigation and data provision: Eiichi Sasaki. Collection of clinical samples: Hidenori Suzuki, Daisuke Nishikawa, Shintaro Beppu, Hoshino Terada, and Michi Sawabe. Review and supervision: Yasuhisa Hasegawa and Masahide Takahashi. Collection of clinical samples, editing and supervision: Nobuhiro Hanai.

## Data Availability

Derived data supporting the findings of this study are not publicly available due to information that could compromise the privacy of research participants.
